# The Impact of Functional Training on Balance and Vestibular Function: A Narrative Review

**DOI:** 10.3390/jfmk9040251

**Published:** 2024-12-03

**Authors:** Eric Bunnell, Matthew T. Stratton

**Affiliations:** Basic and Applied Laboratory for Dietary Interventions in Exercise and Sport, Department of Health, Kinesiology, and Sport, University of South Alabama, Mobile, AL 36688, USA; eb2226@jagmail.southalabama.edu

**Keywords:** functional training, functional movement, vestibular function, balance function, tactical athletes

## Abstract

**Objectives**: The objective of this narrative review was to examine the available literature regarding the increasingly popular method of exercise commonly referred to as “Functional Training” and its potential implications on vestibular and balance function. **Methods**: a narrative review of the experimental literature prior to September 2024 was conducted. To be included in this review, the selected investigations need to include some aspect of vestibular function, balance function, functional training, and/or functional movement and be conducted in humans. **Results**: Evidence emerged to support the claim that implementing a physical fitness training program may improve vestibular and balance function but that a functional training program or a physical fitness program designed to improve functional movements may improve vestibular and balance function to a greater extent. Additionally, these results may be altered by factors such as age and sex. Furthermore, while there is a multitude of literature regarding the impact of functional training on balance, few investigations combine these data with direct assessments of vestibular function. **Conclusions**: Functional training may aid in improving vestibular and balance function, along with scores on common tests such as the Y balance test. However, more research is necessary to elucidate the direct mechanisms by which functional training may impact vestibular and balance function.

## 1. Introduction

Humans have an amazing physical body designed to execute a multitude of life-supporting functions. One such function is the ability to maintain balance. To remain balanced, the body primarily amalgamates information from four systems: the vestibular, visual, somatosensory, and central nervous system [1]. If the body is unable to maintain balance, it can be debilitating and potentially even life-threatening [2]. For example, a decrease in the ability to stay balanced strongly increases fall risk, leading to higher rates of injuries in populations such as older adults and military personnel, negatively impacting quality of life, functional independence, and mortality risk [3,4]. Previous literature suggests that many types of training methods may improve balance function [5,6]. One such method, known as functional training, has grown in popularity in recent years [7].

Functional training (FT) is difficult to define because it is a broad concept with multiple applications and inconsistent implementations and definitions [8]. Furthermore, some authors have argued against such classifications and go so far as to state that FT should not be used to describe any particular training method in part due to this ambiguity [9]. As highlighted in a 2018 review by Stenger, various definitions found throughout the literature contribute to the commonly seen uncertainty of what can truly be deemed as FT. Ultimately, the authors concluded that the definition and subsequent purpose varied based on the specialization of the professional. For instance, Da Silva-Grigoletto et al. [10] discuss that from a physical therapist’s point of view, FT can often be described as neuromuscular conditioning prescribed with the primary purpose of developing and/or maintaining activities of daily living (ADLs) such as bathing and eating. In contrast, Siff [11] defined FT, from a strength and conditioning coach’s point of view, as strength training utilizing methods and movements that will have a high transfer to the movements involved in a given sport or activity. Therefore, training with the purpose of directly aiding sports performance. Which may not always align with the previously mentioned purpose of improvements in ADLs. However, Stenger [8] argued that the simplest definition might come from strength and conditioning coach Mike Boyle, who stated, “Function is essentially purpose. Therefore, FT can be described as purposeful learning” [8]. For the purposes of the present investigation, we will operationally define FT as progressive training utilizing fundamental movements that incorporate both linear and angular accelerations intending to improve functional movement patterns, sports performance, and/or balance function.

Functional movement is a term that has previously been used synonymously with FT [8]. However, they are distinctively different from one another. Functional movement is the ability to perform certain fundamental movements, including the squat and the lunge, while FT is training utilizing said movements within an exercise program for a specific goal [12]. Previous literature suggests that progressively training functional movements, like the Czech get-up and rolling [13] and medicine ball or bodyweight squats [14], may result in greater improvements in balance function than traditional training methods. This is likely why the inclusion of these previously deemed functional movements has become a hallmark of being able to categorize a specific training program as FT. The aforementioned improved adaptations in balance function in response to FT utilizing functional movements in comparison to traditional training methods may be due in part to how many FT movements incorporate both linear and angular accelerations. The peripheral vestibular system contains six semicircular canals (SCCs) that sense angular accelerations and four otolithic organs that sense linear accelerations [15]; because FT incorporates movements that require both angular and linear accelerations, it is reasonable to hypothesize that FT-related changes to the vestibular system may occur. It is also possible that having a better ability to perform the fundamental movements often contained in FT programs (like the squat and lunge) may be in part due to improved balance function [16,17] and potentially vestibular function. If so, FT may provide superior benefits to traditional training for those seeking to improve balance function and/or vestibular function. However, these hypotheses need to be confirmed or refuted by direct investigations. To the authors’ knowledge, the body of literature regarding this relationship and whether there may be differences due to the population in which these relationships are assessed has yet to be fully explored. Thus, the purpose of this narrative review is to examine the currently available literature regarding FT and functional movement and their potential impacts on vestibular function and balance function. Additionally, how this relationship may differ depending on the populations assessed. More specifically, this narrative review will seek to examine any impact FT may have on balance function and vestibular function in young and older adults, as well as tactical populations.

## 2. Literature Search

Literature searches were performed by both authors in PubMed, Google Scholar, Medline, and Scopus using the following terms: “Functional Fitness” AND “Balance Function”, “Functional Fitness” AND “Vestibular Function”, “Functional Training” AND “Balance Function”, “Functional Training” AND “Vestibular Function”, “Functional Movement” AND “Balance Function”, “Functional Movement” AND “Vestibular Function”. The reference and “cited by” sections of the relevant articles were also examined. The inclusion criteria were as follows: published prior to September 2024, be an original investigation that included some aspect of either vestibular function and/or balance function in combination with functional training, functional fitness, and/or functional movement, published in the English language, and conducted in humans. Exclusion criteria included investigations that were not performed in humans, case studies, reviews (narrative, systematic, umbrella, and scoping) and meta-analyses, original investigations that did not state a form of “Functional Training” was employed, and manuscripts that were not written in the English language. Additionally, only peer-reviewed publications were considered. A total of 20 investigations [7,13,14,18,19,20,21,22,23,24,25,26,27,28,29,30,31,32,33,34] were selected for this narrative review. The primary outcomes of interest were alterations in balance function, vestibular function, functional movement ability, and/or plasticity in response to an FT program. Two investigations [28,31] were included in this review that did not directly meet the above criteria, but the authors felt they were necessary for providing the proper context around the potential impact of FT in tactical populations due to the lack of other direct literature. A summary of the selected study methods can be found in Table 1, and the characteristics of the selected study participants are in Table 2.

## 3. Anatomy and Physiology of the Vestibular and Balance Systems

While an exhaustive overview of the anatomy and physiology of the body’s vestibular and balance systems is beyond the scope of this review (for a more comprehensive examination, please refer to Sakka et al. [36], Khan et al. [37], and Horak [1]), a basic understanding of these mechanisms is necessary. The body’s ability to maintain balance is dictated through the merging and processing of information by the vestibular system, vision system, somatosensory system, and central nervous system. Within the vestibular system, there are both peripheral and central components vital to facilitating balance function. The peripheral vestibular system has three main components: the semicircular canals (anterior, posterior, and horizontal), otolithic organs (saccule and utricle), and the vestibulocochlear nerve [15]. The semicircular canals (SCCs) and otolithic organs translate head movements into neural signals, which travel afferently along the vestibulocochlear nerve until they reach the central portion of the vestibular system and brain for processing. Angular accelerations from head movements are sensed by the SCCs, while linear accelerations from head movements are sensed by the otolithic organs. Each SCC is filled with endolymph fluid. At the base of all three SCCs is the ampulla, which contains sensory hair cells on a thick membrane called the cupula.

When the head moves, it forces the endolymph fluid to move inside the SCCs. The movement of the endolymph fluid then causes the hair cells in the ampulla to move toward the tallest of the hair cells, known as the kinocilium. This then creates either an increase (excitation) or a decrease (inhibition) in the afferent firing rate, depending on the canal. The SCCs’ orientation and placement allow them to work in coplanar pairs on both the left and right sides of the head to sense head movements in a three-dimensional plane [15]. For example, a head turn to the right would cause an increase in neural firing in the right horizontal SCC while, at the same time, would cause an inhibition of the left horizontal SCC and a decrease in neural firing. The information is then perceived and interpreted in the brain as a horizontal head turn to the right.

The orientation and placement of the otolithic organs allow them to sense horizontal (utricle) and vertical (saccule) linear head movements [15]. Inside both these organs are thousands of otoconia made up of calcium carbonate. The otoconia rest in a sticky structure called the maculae and, like the SCCs, are surrounded by endolymph fluid. When the head moves, gravity forces the otoconia to move and increases the density of the macula, which creates an inertial mass that stimulates the hair cells of the otolith organs near the striola (the central dividing line of the maculae). This movement causes a polarization in neural firing rates when the hair cells move. Like the SCCs, the stereocilial movement toward the tallest stereocilia (kinocilia) creates an increase in neural firing rate, and movement away from the kinocilia creates a decrease in neural firing rate [15]. An example of saccule stimulation would be a head tilt backward, causing an increase in the neural firing rate toward the dorsal section of the striola while at the same time causing a decrease in the neural firing rate toward the ventral section of the striola. The information is then perceived and interpreted as a backward head tilt.

Beyond the peripheral portion is the central portion of the vestibular system. It is comprised of the central vestibular nucleus complex (CVNC). Within the CVNC, the vestibulocochlear nerve begins in the brainstem, where nuclei are gathered and connected back to the SCCs and otolithic organs [1]. The CVNC has two sections that branch out from the right and left sides of the brainstem and receive information from multiple sources. For example, on both sides of the brainstem, there are four specific nuclei (inferior, lateral, medial, and superior vestibular nucleus) that receive sensory input afferently from the peripheral portions of the vestibular system, merge the information and then pass the information to the cerebellum, cortex, thalamus, reticular formation, visual system, and spinal cord to coordinate vestibular reflexive movements.

The three primary vestibular reflexes are the vestibulo-ocular reflex (VOR), the vestibulocollic reflex (VCR), the vestibulospinal reflex (VSR). The VOR is a reciprocal connection between the vestibular and visual system that fixates gaze and helps focus vision on an object while the head is in motion to help the body maintain balance [38]. The reciprocal connection of this reflex causes compensatory eye movements that are equal in magnitude and opposite in the direction of head movements. For example, a head turn to the left causes rightward eye movement. The VCR, or “head righting reflex,” is the response of the medial vestibulospinal tract to vestibular stimulation when the head moves off-center in relation to the body. The neck muscles, in particular the sternocleidomastoid muscle (SCM), are responsible for controlling this reflex as they contract and relax to move the head in the opposite direction the vestibular system senses the body is falling [39]. For example, if there is a feeling of falling backward, the neck and head move forward to keep the head upright and help the body maintain balance. The VSR is the reciprocal connection between the vestibular system and the spinal cord to maintain an upright posture. VSR is often discussed in terms of postural control or amount of standing sway and relates to whole-body balance ability through the integration of several sensory systems. Balance ability, often as measured through assessment of the VSR, is the reflex that has been found most often to benefit from physical training programs [40]. A deeper exploration of this reflex is thus warranted.

**Figure 1:**
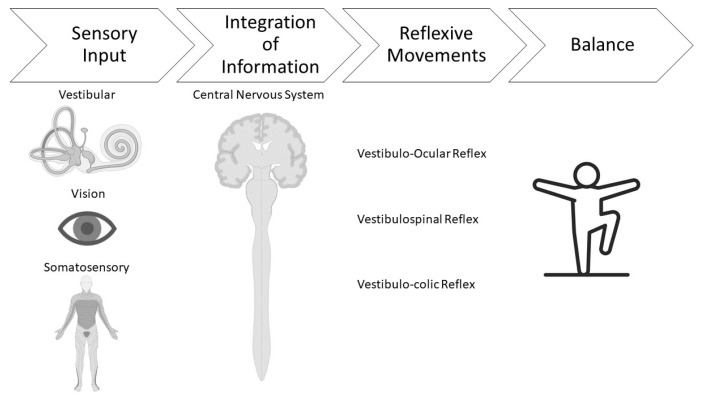
An overview of system integration leading to the regulation of balance function in healthy individuals.

During the VSR, integrated information coming from the vestibular and spinal cord sensory inputs helps keep the body upright and positioned over the center of gravity [37]. The somatosensory systems provide tactical sensory input for the VSR. This information arises from tactile perception (touch), which creates awareness of objects in the environment and the body’s position in space [38]. Proprioceptive receptors are part of the somatosensory system and include structures like muscles and joints. These receptors allow the body to know where it is in space without having to visualize it. For example, lifting a leg in front of the body can still be perceived, even with eyes closed. The VSR pathway from the somatosensory and proprioceptive systems travels to the central vestibular nucleus complex and integrates with information from the peripheral vestibular and visual systems. The vestibulospinal system then sends information back to the spinal cord, so postural reflexes, head movement, and changes in axial musculature work together to maintain an upright posture [1]. The two main descending pathways are the lateral and medial vestibulospinal tracts. The lateral vestibulospinal tract interacts with four specific vestibulospinal reflexes: the ankle strategy, the hip strategy, the suspensory strategy, and the stepping strategy. The ankle strategy is when the leg muscles contract to align the trunk of the body with the knees and hips. It is used primarily for smaller disturbances in balance. The hip strategy occurs when the muscles in the leg contract and the hip and the head move in opposite directions to redistribute the weight of the body. The suspensory strategy is when the knees, ankles, and hips flex to lower the body’s center of gravity. The stepping strategy is a corrective step taken when large corrections are needed when the center of gravity has moved outside the base of support. These four strategies work together to maintain balance when the center of gravity moves outside the base of support [38]. An overview of the integration of these systems and their impact on balance function can be seen in Figure 1.

### 3.1. Plasticity of the Vestibular and Balance Systems

Plasticity is part of the brain’s way to learn, remember, reorganize, and recover from injury [41]. A more commonly accepted explanation of plasticity is when the brain’s neural networks change, grow, or rewire to function differently than they did before. Changes to the vestibular system, vision system, somatosensory system, and/or central nervous system as a result of aging, injury, or pathology could have a significant effect on a person’s quality of life and ability to maintain balance [42]. Structural brain plasticity in the hippocampus is critical for balancing, as made evident by a 2011 investigation by Hüfner and colleagues [18]. Fourteen semiprofessional to professional dancers (average: 16.9 yrs experience) and seven slackliners (average: 2.8 yrs experience) were assessed via MRIs, memory-influencing reading tests, spatial tests, and visual discrimination tasks. When compared to age and sex-matched (~24.9 y/o, 10 males and 11 females) controls who did not have expert training in dancing or slacklining, the trained dancers and slackliners displayed increased hippocampal formation volumes. However, it must be noted that the characterization of the participants was based on self-reported training histories completed at the time of enrollment. As such, as with any data that primarily characterized participants based on self-report, they should be interpreted with care. Although, to lend credence to the aforementioned findings, a 2019 investigation by Giboin et al., found that 6-weeks of slackline training in balance training naïve adults resulted in functional connectivity alterations in the brain regions associated with posture and balance control suggesting balance training related improvements in large scale plasticity [21]. However, interestingly, balance performance was not different in non-slackline-specific balance tests. This led the authors to conclude that although improvements in plasticity may occur, they are likely to be largely specific to the task performed during training. These data may help elucidate as to how FT may aid improvements in functional movements, by aiding plasticity. Previous literature also suggests that plasticity takes place in both the peripheral and central portions of the vestibular system as a response to a change in activity like the loss of vestibular input or gravity conditions during orbital flights [43], like those commonly undertaken by tactical populations such as military pilots. Additionally, by inducing structural changes in plasticity, both the peripheral and central portions of the vestibular systems may benefit from physical activity [35].

Physical activity has been examined as a way to improve the long-term ability of the brain to change to challenging dynamic environments [35]. Whole-body exercises that demand the body’s ability to maintain balance, such as dancing and figure skating, evoke specific structural and functional changes in the brain [18,44]. Because the vestibular system is active during self-motion activities, vestibular inputs may play an important role in exercise-induced neuroplasticity [45]. Rogge et al. [35] conducted a study with 37 healthy older adults (average age: 69 yrs) to see if balance training and challenging the sensory-motor system could induce structural plasticity. The participants were randomly placed into one of two groups: a balance training group or a relaxation training group. They trained in their groups for 50 min twice weekly over a period of 12 weeks. The balance training involved maintaining balance on varying surfaces like foam, wobble boards, and perturbation platforms. The relaxation training involved tensing small muscle groups (hands, shoulders, feet, legs, etc.) and then relaxing those same muscle groups. After 12 weeks, participants were assessed again with a dynamic balance assessment and a structural MRI to measure cortical thickness. The authors demonstrated that greater gains in balance performance correlated to an increase in cortical thickness. Additionally, improved balance performance led to a decrease in gray matter volume of the left putamen. These findings suggest that increased physical activity and increased balance function may induce structural plasticity in the central nervous system in areas related to balance, such as the integration of spatial information and visual-vestibular self-motion processing.

### 3.2. Functional Training and Vestibular and Balance Function

As previously mentioned, physical activity has been shown to promote structural plasticity in the vestibular systems and improve the ability to maintain balance by influencing both the higher-level CNS (as examined by changes in cortical thickness) [21,35] and the lower-level and more peripheral systems, which can be seen through an examination of the vestibular reflexes (i.e., VOR, VSR, and VCR) [43]. It is reasonable to hypothesize that FT may follow the same pattern regarding vestibular plasticity and maintaining balance. Riemann et al. conducted a study to compare the balance performance between 48 national-level Olympic weightlifters and 42 competitive distance runners, all between the ages of 40 and 60 [19]. Olympic weightlifting was chosen as it comprises two events, the “snatch” and “clean and jerk”, which require a large amount of power and coordination of the entire body to raise a barbell from the floor to over the head. Additionally, these two exercises are very commonly utilized within FT methodology. The runners regularly trained up to 30 km a week and primarily raced 10 Ks to marathons. Participants were asked to complete a balance test with four different conditions on both legs for 30 seconds: 1. balance on a firm surface with eyes open, 2. balance on a firm surface with eyes closed, 3. balance on a foam surface with eyes open, and 4. balance on a foam surface with eyes closed. The authors found that Olympic weightlifters had significantly better balance when compared to those in the running group, especially in conditions with their eyes closed. These results suggest that these exercises commonly found in FT routines may provide better training for balance function than running alone. However, it is important to note that direct measures of vestibular function were not conducted; therefore, the extent to which these alterations are attributable to changes in vestibular function remains uncertain. Additionally, these results were cross-sectional in nature, making it challenging to draw direct conclusions about how such training may impact long-term changes in vestibular function and balance.

In one of the sole investigations to assess longitudinal changes to both vestibular function and balance in response to FT, Fu et al. [30] conducted a study among a total of 101 5–6-year-old children from Tianjin, China. The children were divided into either an experimental (FT) or a control group (kindergarten-based fitness curriculum) for 12 weeks. The functional training children’s program focused on age-appropriate motor development skills, while the control group’s program focused on group games and free play. After the 12-week intervention, the children’s gross motor skills were evaluated using the Test of Gross Motor Development-2, which includes 12 fundamental movements, such as running and hitting a stationary ball. Their fitness levels were assessed through the National Physical Fitness Measurement, which comprised activities like running, balancing, and stretching. Additionally, their sensory integration was measured using the Child Sensory Integration Scale, which encompasses four different areas: vestibular function, tactile defensiveness, proprioception, and learning ability. It is important to note that the vestibular function domain was evaluated by measuring postural control and balance ability. The FT group showed increased flexibility, balance, gross motor control, and vestibular function when compared to controls. These data begin to support the idea that FT may help improve vestibular function along with the previously noted improvements in balance function. However, more investigations are needed in other populations and in comparison to more variations in training to further elucidate the degree to which improvements in balance function may be related to concomitant improvements in vestibular function.

### 3.3. Functional Training Variations

As previously mentioned, the term functional training is utilized in a multitude of contexts that vary in their definition and, ultimately, purpose and goal. Consequently, the different approaches to FT are also difficult to define because of the variations in which training may resemble that described in some contexts as FT but under different names [8]. For instance, a systematic review by Rice and colleagues concluded that power training, although not labeled as functional, many of the movements and programs in the studies included resembled FT, helped improve functional performance in older adults in 10 out of 12 studies reviewed when compared to traditional resistance training [46]. To the author’s knowledge, the body of literature regarding other common variations, sometimes known as self-guided FT and targeted FT, as well as their effects on vestibular and balance function, are yet to be fully explored. One study found that self-guided training, though not explicitly labeled functional, was beneficial in improving postural control in older adults, while guided training only helped reduce fall risk [47]. Lastly, a review of current studies found that implementing a common variant known as high-intensity FT (HIFT) can be used to target improvement in sport-specific performance [48]. Although, due to the nature of these variants falling outside of our working definition of FT due to either not being labeled as targeting functional movement (e.g., self-guided, power, targeted) or the highly variable nature that lacks the necessary structure and progression as described by Da Silva-Grigoletto et al. [10] to truly be deemed functional (e.g., HIFT) we will not be including them in the following sections.

### 3.4. Population Specific Effects

#### 3.4.1. Young Adults

It is well understood that regularly participating in fitness programs plays a vital role in health across the lifespan. Crimmins et al. [49] suggested that to have a better chance at a long and healthy life, fitness programs need to be implemented at younger ages and not just for older adults or at end-of-life scenarios. Questions still exist regarding how effective FT is compared to other traditional resistance training in regard to improving or altering balance function in young adults. Weiss et al. conducted a study in 2010 to examine the impact of 7 weeks of FT or traditional resistance training on muscle strength, endurance, flexibility, and balance in a group of 38 young adults aged between 18–32 years [22]. The participants were randomly assigned to either a control group (traditional resistance training) or an experimental group (FT). Throughout the 7 weeks of training, the control group performed exercises like the bench press and seated row, while the experimental group performed exercises like barbell back squats and multidirectional lunges. Balance results revealed that both groups improved on one-leg balance tests after training, but there were no significant differences between the training modalities. Similarly, a 2009 investigation by Kibele and Behm found comparable improvements in dynamic balance tests from both types of programs [23]. However, since both studies recruited untrained populations, it is possible that the lack of prior training experience may have influenced the results. In essence, with no prior training, nearly any approach could create a sufficient stimulus to yield considerable progress. Similarly, a 2023 investigation by Khazaei et al. recruited well-trained participants for both groups, yet significant differences between groups (traditional and FT) in balance results were not found [25]. This may suggest that in addition to training status being a factor that should be considered when interpreting the present data, age may play a role as well. It is possible that young adults may have similar functional movement abilities, which could explain why their balance improved after training. However, this may not hold true throughout the lifespan. Additionally, the degree to which these alterations in balance are due to impacts on vestibular function is unknown.

#### 3.4.2. Older Adults

As we age, functional movement abilities decline [50], stressing the importance of performing FT to maintain and improve the ability to balance the body. Although many types of exercise interventions claim to be effective at improving functional abilities in older adults, there is still a debate as to which methods are best. Morucci et al. performed a study in which they assessed the effects of a 24-week exercise program on functional fitness in 18 healthy older adults (average age: 72.8 yrs) who regularly participated in a fitness class at their local gym [29]. All participants received pre- and post-training evaluations of functional tests to measure strength, dynamic balance, flexibility and a self-perceived questionnaire about the indirect effects of physical activity on their quality of life. Training sessions lasted about 60 min and were held twice a week. Training consisted of FT exercises for both the lower and upper body, focusing on multimodal movements. The authors found an improvement in dynamic balance, flexibility, and strength. However, it must be noted that this was a single-arm investigation that lacked a traditional training or control group. As such, these data suggest that performing FT as an older adult may improve the ability to maintain balance. However, it does not demonstrate if there is an advantage to performing FT over traditional strength training and how this might impact vestibular function. However, as the vestibular system is highly involved in the regulation of balance function, it is reasonable to hypothesize concomitant adaptations occurred, but direct tests are needed before it can be said for certain.

Other studies also support the claim that FT may improve balance function but suggest there may be differences between sexes [26]. A 2013 investigation by Pacheco and colleagues aimed to compare traditional training to FT in a group of 101 middle-aged (40–60 yrs) and older adults (>60 yrs) by assessing Functional Movement Scores (FMS) and the Y-Balance Test. The FMS is a battery of tests that evaluates balance, flexibility, and stability. It includes a deep squat, hurdle step, inline lunge, shoulder mobility, leg raise, rotary stability, and trunk stability. The Y-Balance Test evaluates balance, lower body strength, and flexibility by having the participant balance on one leg while the other leg extends out as far as possible and touches the floor in three different directions (anterior, posterolateral, and posteromedial). The participants were divided into two groups: an FT group and a conventional training group. Both groups trained twice a week over 12 weeks for a total of 24 sessions. Once again, the FT group performed exercises like squats and lunges, while the conventional training group performed exercises such as bench presses and leg extensions. FMS improved significantly in both groups. However, males in the FT group demonstrated significantly greater improvements in their FMS scores than their female counterparts. Results also revealed that females in the FT group significantly improved FMS more than those who performed conventional training. Furthermore, only the FT group showed improvement on the Y-Balance Test. These results indicate that both FT and conventional training may aid functional movements. However, FT may also have additional benefits for improving balance function. However, the benefits may differ between sexes and the individual test utilized.

#### 3.4.3. Tactical Populations

Tactical populations–such as the military, police, firefighters, and combat athletes–all need to have a certain level of fitness to meet the demands of their physical jobs or sports. For example, a soldier in the military moves under, over, and around objects in combat gear while engaging enemy targets. For this, a soldier needs to be physically fit and able to maintain balance in order to avoid injury and death and defeat the enemy. Readiness is assessed differently depending on the population in question. A Soldier needs to be able to pass the Army’s new fitness test, the Army Combat Fitness Test (ACFT) [51]. In many fire departments, firefighters prove their readiness by passing the Candidate Physical Ability Test (CPAT), and police need to pass the Physical Ability Test (PAT). The best type of training to prepare tactical populations for these tests is still yet to be determined.

Unfortunately, few investigations have been conducted in military populations to ascertain if any benefits exist for implementing FT over traditional training methodologies for improvements in balance function. While not directly assessing measures of balance or vestibular function, a still enlightening 2023 study conducted in Finland by Helen and colleagues compared FT to traditional military physical training [31]. A group of 133 servicemen between the ages of 18 and 28 was split into two groups: an experimental group that undertook a functional resistance training program that included exercises like the squat, deadlift, kettlebell swings, and burpees, and a control group that performed their traditional military physical training protocol primarily consisting of exercises like running and calisthenics. Following 19 weeks of training, strength, and endurance significantly improved in the FT group when compared to the control group. Endurance improved by 11.6%, as observed in the 12-minute run test. Strength improved by 3.8% for the upper body and 5.0% for the lower body, as assessed by the maximal voluntary isometric contractions (MVICs). These findings suggested that FT is an effective approach to improving Soldiers’ fitness and may provide additional benefits over traditional training methods. However, when examining the effect sizes for the given variables, only the 12-min run improvement approached a large effect. However, even the small effects noted for alterations in upper and lower body muscular performance may provide benefits to warfighters who may potentially be in life-threatening combat situations. These data, combined with previous findings, suggest that when compared to either traditional resistance or military training, FT may provide greater benefits to improvements in muscular strength, aerobic endurance, speed, flexibility, and muscular endurance [24,28]. Furthermore, as these results are promising for other metrics of performance, future investigations should seek to ascertain if similar benefits would extend to measures of balance and vestibular function in these tactical populations.

Similar to military and first responder populations, many combat athletes are put under high-stress situations that require quick reaction times [25], high levels of balance and agility [52], and the ability to display increased levels of anaerobic power [53], which are common targets of the FT methods previously described. Khazaei and colleagues randomly placed 17 elite (1st to 3rd place in national or international competitions within the 5 years prior to enrollment) taekwondo athletes into groups undergoing 8 weeks of either FT (n = 9) or traditional resistance training (n = 8). While both groups significantly improved at the end of the 8 weeks, no differences were seen for changes in measures of strength, flexibility, or dynamic balance as assessed via the Y balance test between the two groups at the end of the 8 weeks. However, the FT group did display improved average and mean power across a 30 s Wingate anaerobic power test. These data suggest that for elite athletes of sports already comprising high levels of balance and coordination, like many combat athletes, FT may not improve balance function to a significant degree when compared to traditional training after only 8 weeks. However, other benefits may still be seen over traditional resistance training that may aid competition performance (e.g., anaerobic power). Together, these studies demonstrate some of the beneficial effects of FT, and though these studies did not specifically measure vestibular function, based on previously observed findings [27,30], it is reasonable to hypothesize that vestibular function may improve alongside balance function. However, once again, direct investigations are needed to either confirm or refute this hypothesis.

### 3.5. Functional Movement and Vestibular and Balance Function

Although FT has been shown to improve vestibular and balance function [30], there are still those who conclude that FT only improves the specific movements practiced and does not translate to better functional movement overall [20]. To examine this hypothesis, Mahdieh et al. conducted a study to see if Dynamic Neuromuscular Stabilization (DNS) exercises would improve measures of functional movement more than traditional physical fitness exercises [13]. DNS exercises are typically used to restore or rehabilitate functional movements. DNS therapy starts very basic with infant movements like rolling and oblique sitting, then advances as the individual proves proficiency in movements like squats and the Czech get-up. The participants (34 healthy non-athlete females) were split into two groups and implemented the different training protocols for 6 weeks (50-minute sessions, three times per week). Both groups significantly improved on the FMS, but a significant group interaction effect revealed the DNS group had a significantly greater improvement on the FMS than the physical fitness group did following the 6 weeks of training. However, the effect size varied for each functional movement in the FMS, suggesting the need for more specific exercises to complement the functional movement. Furthermore, direct measures of vestibular function were not assessed. Future investigations should seek to examine the degree to which these benefits in functional movement proficiency are due to alterations in vestibular function.

## 4. Limitations and Future Directions

As with all research, the present review is not without limitations. Firstly, despite the effort to broadly search for available literature, it is likely that some investigations may have been omitted due to not being connected to the selected search keywords. A significant limitation that cannot be overlooked is the lack of data directly examining alterations in vestibular function in response to FT. However, a goal of this narrative review was to highlight the potential mechanistic connections between balance function and vestibular function and the lack of literature surrounding their concomitant responses to FT so that future investigations may help shed more light on this particular topic. To specifically assess vestibular function, future investigations could include the use of the video Head Impulse Test and the Ocular Vestibular Evoked Myogenic Potential for the vestibulo-ocular reflex, the cervical Vestibular Evoked Myogenic Potential for the vestibulo-collic reflex, and the Modified Clinical Test of Sensory Interaction and Balance for the vestibulospinal reflex. The large variation in protocols used and what each research group determined FT and traditional training may have impacted the results of this particular review. Thus, it is recommended that future investigations into this topic develop clear guidelines as to what may truly be considered FT to aid in future reviews of this nature. Lastly, the lack of data on certain subgroups (e.g., moderately trained, various tactical settings) makes drawing a firm conclusion on the impact of FT on balance function and vestibular function in these populations difficult.

## 5. Conclusions

Functional training is an increasingly popular training methodology that seeks to improve not only physical fitness but foundational movement patterns, such as the squat or lunge. The primary findings of this narrative review are that there is evidence to claim that implementing a physical fitness training program may improve balance function but that an FT program or a physical fitness program designed to improve functional movements may improve balance function to a greater extent. Additionally, these positive changes in balance function may represent improvements or, at the very least, coincide with enhancements in vestibular function [30]. However, data directly assessing alterations in vestibular function that may concomitantly occur with these changes in balance function in response to functional training were not able to be found to be included in this review. Furthermore, a multitude of factors may impact these findings. For instance, differences in balance function appear to be minor when younger, untrained, or elite combat populations undergo either traditional or functional training programs. Conversely, in older populations, functional training may prove advantageous over traditional methodologies in improving scores on common tests such as FMS and the Y balance test. As such, population considerations, such as age and training history (general and balance specific), should be made when either designing future investigations or interpreting the present literature. Future investigations should seek to elucidate the degree to which alterations in vestibular function in response to divergent training methodologies aid the long-term improvement or maintenance of balance function. Furthermore, how this may impact differing and underrepresented populations such as those in tactical settings.

## Figures and Tables

**Table 1:** Selected study characteristics.

Reference	Length	Intervention	Variables Assessed	Findings
***FT and Plasticity***				
Hufner et al., 2011 [18]	Cross-sectional	MRI in trained dancers and slackliners (n = 21) or age and sex-matched controls (n = 20)	Memory-influencing reading tests, spatial tests, and visual discrimination tasks	Trained subjects displayed larger hippocampal formation volumes, suggesting increased structural brain plasticity.
Giboin et al., 2019 [21]	6-weeks	45 min of slackline training 2× per week (n = 17) or control (n = 13)	fMRI, balance, and H-reflex	Slackline training improved neuroplasticity. However non-slackline balance tests did not change compared to controls.
Rogge et al., 2018 [35]	12-weeks	50 min 2× per week of either balance training (n = 19) or relaxation training (n = 18)	Structural MRI and dynamic balance	Increased cortical thickness and balance performance following balance training over relaxation training
***FT and Vestibular Function***				
Fu et al., 2022 [30]	52-weeks	Two 50-minute functional training sessions per week (n = 51) or one 50-minute kindergarten-based fitness session per week (n = 50)	Test of Gross Motor development-2, National Physical Fitness Measurement, Child Sensory Integration Scale	The functional training group performed better on vestibular tests post-training compared to the kindergarten-based Fitness Group
***FT on Balance in Young Adults***				
Weiss et al., 2010 [22]	7-weeks	3× per week of either Traditional RT (n = 19) or Functional RT (n = 19)	ME (Pushups and sit-ups), 1RM (Bench Press and Squat), BC (weight and circumferences), One-leg Balance	Functional training improved flexibility and balance to a greater degree than traditional training.
Kibele et al., 2009 [23]	7-weeks	Unstable RT (n = 20) or Stable RT (n = 20)	Static and dynamic balance, 1 leg hopping, leg extension strength, sit-up endurance	Unstable RT increased sit-up and 1-leg hopping performance over stable RT
***FT on Balance in Older Adults***				
Riemann et al., 2020 [19]	Cross-sectional	30-s 2-leg balance tests on firm and foam surfaces with eyes open or closed in weightlifters (n = 48) or runners (n = 42)	MLCPV throughout each condition	Olympic Weightlifters performed better than runners in both eyes closed conditions
Guler et al., 2021 [7]	8-weeks	Conventional RT (n = 20) or Functional RT (n = 26)	FMS total score, balance, and BC	Functional training significantly improved balance, FMS, and BC. No changes from pre to post were seen in the conventional training group
Sedaghati et al., 2022 [34]	8-weeks	60 min 3× per week of either FT (n = 14) or conventional care (n = 14)	Borg balance assessment and SPPB	Significant improvements in all measures in the FT group over conventional care.
Pacheco et al., 2013 [26]	12-weeks	75 min 2× per week of either conventional RT (n = 50) or functional RT (n = 51)	Y-balance Test, FMS score	FMS improved equally in both groups. Males improved more than females.
Emilio et al., 2014 [27]	12-weeks	50 min 2× per week of either proprioceptive Training (n = 20) or usual physical activity (n = 24)	The assessment of tender points, chair sit and reach test, hip joint mobility, the get-up and go test, blind flamingo test, lumbar strength, Berg balance scale, Tinetti scale	The proprioceptive training group had significantly higher values compared to the usual activity group in lower body flexibility, dynamic balance, and lumbar strength
Morucci et al., 2022 [29]	24-weeks	60 min 2× per week of functional training (Single-arm investigation)	Functional performance (grip strength, chair sit to stand, timed get up and go, sit and reach, and back scratch)	Significant improvements were seen in flexibility (sit and reach and back scratch) and balance (timed get up and go)
***FT on Balance and Functional Movement in Tactical Populations***				
Gross et al., 2009 [33]	6-weeks	75 min of functional training (Single-arm investigation)	FMS, agility, single leg hop time and distance, VJ height, and BC	All scores improved in relation to baseline
Khazaei et al., 2023 [25]	8-weeks	75–90 min sessions 3× per week of either Functional Training (n = 9; FT) or Traditional Resistance Training (n = 8; TRT)	The sit and reach test, the Y-balance test, the Wingate test, a power test, a speed test, an agility test, coordination tests, the Bruce test, and the V02 max test	No significant differences observed between groups. Both groups improved in aerobic power, muscle power, speed, agility, reaction time, lower body strength, upper body strength, core endurance, and upper body muscle endurance
***FT on Performance in Tactical Populations***				
Reau et al., 2018 [28]	16-weeks	90 min of functional training 4× per week (n = 148) (single arm)	1.5-mile run, maximum plank time, max pullups, max pushups, BW squats within a minute	Performance improved in 89% of participants, resulting in a 39% reduction in days missed due to illness
Helen, et al., 2023 [31]	19-weeks	75 min 2× per week of functional training (n = 66) or 66 min 2× per week of traditional RT (n = 67)	12-minute run, Upper and Lower body power, BC, and maximum number of pushups	All performance metrics improved in the FT group, while no changes were seen in the traditional RT group
***FT and Functional Movement***				
Wright et al., 2015 [20]	4-weeks	30 min 4× a week of either movement-based (intervention; n = 11) or generic multi-sport (control; n = 11) exercise	FMS (total score)	The intervention made a small impact on the FMS total score but did help with core stability
Mahdieh et al., 2020 [13]	6-weeks	50 min 3× per week of either dynamic neuromuscular stabilization (DNS; n = 19) or physical fitness (PF; n = 15) training	Single-leg squat, Y-Balance test, Functional Movement Screener (FMS), landing error scoring system	Both groups improved on the balance test, but the DNS group improved significantly more than the PF group
Yildiz et al., 2019 [14]	8-weeks	65 min 3× per week of either Functional Training (FTG; n = 10) or Traditional Training (TTG; n = 10) or Control (CG; n = 8)	Flexibility, CMJ, 10 m acceleration, T-test agility, right dynamic balance, left dynamic balance, static balance, FMS (total score)	FMS scores of FTG increased while those of the CG and TTG decreased following the 8 weeks
Song et al., 2014 [32]	16-weeks	60 min 3× per week of either FT (n = 31) or traditional training (control; n = 31)	Strength, flexibility, and FMS (total score)	All variables improved to a greater extent following FT when compared to the control group

RT: Resistance Training; fMRI: Functional Magnetic Resonance Imaging; MLCPV: Average mediolateral center of pressure velocity; ME: Muscular Endurance; BC: Body Composition; FMS: Functional Movement Screen; HIFT: High-Intensity Functional Training; APFT: Army Physical Readiness Test, DNS: Dynamic Neuromuscular Stabilization; PF: Physical Fitness; CMJ: Countermovement Jump; FTG: Functional Training Group; TTG: Traditional Training Group; CG: Control Group; FT: Functional Training; TRT: Traditional Resistance Training; SPPB: Short Physical Performance Battery.

**Table 2:** Selected study participant details.

Reference	Participants	Age (yrs; Mean ± SD)	Notes
***FT and Plasticity***			
Hufner et al., 2011 [18]	Trained: N = 21 (10 M, 18 F) 7 ballet dancers 7 ice dancers 7 slackliners Controls: N = 20 (8 M. 12 F)	Trained: 24.9 ± 7.8 Control: 26.7 ± 7.8	Trained participants performed ≥3 h of training per week. Controls still performed leisure sports but did not regularly perform dance or slacklining activities
Giboin et al., 2019 [21]	Training: N = 17 (10 M, 7 F) Control: N = 13 (3 M, 10 F)	Training: 25 ± 4 Control: 22 ± 2	All participants needed to be right-leg dominant and naïve to any balance training
Rogge et al., 2018 [35]	Balance: N = 19 (7 M, 12 F) Relaxation: N = 18 (7 M, 11 F)	Balance: 43.9 ± 14.9 Relaxation: 46.1 ± 15.4	Participants were required to not regularly perform physical activity (>5 exercise sessions per month during the last 5 years)
***FT and Vestibular Function***			
Fu et al., 2022 [30]	Functional Training: N = 51 (25 M, 26 F) Kindergarten-based Fitness N = 50 (26 M, 24 F)	5–6 years old. Direct group ranges were not reported	All participants were 5–6-year-old children in Tianjin, China
***FT on Balance in Young Adults***			
Weiss et al., 2010 [22]	Traditional RT N = 19 Functional RT N = 19 Sex distributions were not reported	18–32 Group averages and SD were not reported	Active (definition not reported) and low to moderate risk according to 2006 ACSM risk stratification guidelines
Kibele et al., 2009 [23]	Unstable RT N = 20 Stable RT N = 20 28 M 12 F Group sex distributions were not reported	Males 23 ± 2.4 Females 22 ± 1.8 Group averages were not reported	All participants were required to be untrained and be right-leg dominant
***FT on Balance in Older Adults***			
Riemann et al., 2020 [19]	Olympic Weightlifters N = 48 (26 M, 22 F) Runners N = 42 (25 M, 17 F)	Olympic Weightlifters M: 48.8 ± 9.7 F: 45.7 ± 8.1 Runners M: 47.8 ± 7.5 F: 47.5 ± 10	Olympic weightlifters were recruited from the 2017 National Masters Olympic Weightlifting Competition, and runners were required to train ≥30 km per week and primarily train 10k to marathon distances
Guler et al., 2021 [7]	Conventional RT N = 20 Functional RT N = 26 22 M 24 F Group sex distributions were not reported	Conventional RT 51.6 ± 3.7 Functional RT 52.8 ± 4.0	All participants were required to exercise at least 3× per week prior to enrollment
Sedaghati et al., 2022 [34]	Functional Training N = 14 Control N = 14 All Males	70.8 ± 2.5 Ground averages were not reported	All participants were untrained but otherwise healthy and able to perform daily activities without assistance
Pacheco et al., 2013 [26]	Functional Training N = 51 Conventional Training N = 50 45 M 56 F Group sex distributions were not reported	Males 56.2 ± 9.7 Females 53.6 ± 7.9 Group averages were not reported	All participants were enrolled in a physical activity program at the University of Sao Paulo
Emilio et al., 2014 [27]	Proprioceptive Training N = 20 (11 M, 9 F) Usual Physical Activity N = 24 (14 M, 10 F)	Proprioceptive Training 79.4 ± 7.4 Usual Physical Activity 77.0 ± 6.9	Healthy older adults who were able to perform regular physical activity unassisted
Morucci et al., 2022 [29]	N = 18 (4 M, 14 F)	Full Group 72.8 ± 7.5 Males 72.6 ± 9.1 Females 72.9 ± 7.4	Physically independent older adults (>60 years of age)
***FT on Balance and Functional Movement in Tactical Populations***			
Gross et al., 2009 [33]	N = 90 (80 M, 10 F)	Full Group 35 ± 5.0	All participants were active-duty soldiers seeking to return to active duty following injury
Khazaei et al., 2023 [25]	Functional Training N = 9 Traditional Training N = 8 All Female	Functional Training 21.1 ± 2.9 Traditional Training 22.3 ± 3.1	Taekwondo athletes who had placed 1st to 3rd place in national or international competitions over the last 5 years
***FT on Performance in Tactical Populations***			
Reau et al., 2018 [28]	Functional Training N = 148 All Male	Age was not reported	Retrospective analysis of active duty firefighters who participated in a functional training course
Helen, et al., 2023 [31]	Functional Training N = 66 Traditional RT N = 67 All male	Functional Training 19 ± 1.0 Traditional RT 19 ± 1.0	All participants were military conscripts in the Finnish Defense Forces
***FT and Functional Movement***			
Wright et al., 2015 [20]	Movement-based Group N = 11 Generic Multi-Sport Group N = 11 Sex was not reported	Movement-based Group 13.0 ± 0.8 Generic Multi-Sport Group 13.8 ± 0.8	Participants were students from the “gifted and talented” program from local secondary schools
Mahdieh et al., 2020 [13]	DNS N = 19 PF N = 15 All Female	DNS 18.8 ± 0.7 PF 18.9 ± 0.9	Non-athlete, generally healthy female students
Yildiz et al., 2019 [14]	Functional Training N = 10 Traditional Training N = 10 Control N = 8 All male	Full Sample 9.6 ± 0.7 Group averages were not reported	At least 2 years of previous tennis experience and had an FMS score of <15 at pre
Song et al., 2014 [32]	Functional Training N = 31 Traditional Training N = 31 All male	Functional Training 17.0 ± 1.1 Traditional Training 16.7 ± 0.9	Elite high school basketball players. The definition of elite was not reported

RT: Resistance Training; FMS: Functional Movement Screen; FT: Functional Training; DNS: Dynamic Neuromuscular Stabilization; PF: Physical Fitness.
